# Two rare diseases, acute calcific retropharyngeal tendinitis, and crowned dens syndrome, mimicking meningitis: A case report

**DOI:** 10.3389/fneur.2022.946222

**Published:** 2022-10-21

**Authors:** Eriko Igami, Jiro Fukae, Kazo Kanazawa, Atsuhito Fuse, Asuka Nakajima, Hiroto Eguchi, Nobutaka Hattori, Yasushi Shimo

**Affiliations:** ^1^Department of Neurology, Juntendo University Nerima Hospital, Tokyo, Japan; ^2^Department of Research and Therapeutics for Movement Disorders, Juntendo University School of Medicine, Tokyo, Japan; ^3^Department of Neurology, Juntendo University School of Medicine, Tokyo, Japan

**Keywords:** neck pain, fever, acute calcific retropharyngeal tendinitis, crowned dens syndrome, cervical computed tomography

## Abstract

We report two rare cases. One involved acute calcific retropharyngeal tendinitis, an inflammatory condition of the longus colli tendon triggered by the deposition of calcium hydroxyapatite crystals. The other involved crowned dens syndrome, caused by pseudogout of the atlantoaxial junction following deposition of calcium pyrophosphate dehydrate or calcium hydroxyapatite. Although these two diseases involve different mechanisms, the common symptoms of neck pain and fever resemble those of meningitis. Accurate diagnosis can thus be difficult without background knowledge of these conditions. Cerebrospinal fluid examination and cervical computed tomography are useful for distinguishing these pathologies from meningitis.

## Introduction

Meningitis is defined as inflammation of the meninges, usually caused by a central nervous system infection (1, 2). However, on rare occasions, meningitis can be caused by neoplasms, drugs, or autoimmune disease (3–5). The classic triad of meningitis comprises fever, neck stiffness, and altered consciousness. The prevalence of symptoms in meningitis is 87% for headache, 83% for neck stiffness, 77% for fever, and 69% for altered consciousness (6). Only 44% of episodes are characterized by the classic triad of fever, neck stiffness, and altered consciousness (6). At least two of the four signs of the classic triad plus headache are present in 95% of patients with meningitis (6). Acute calcific retropharyngeal tendinitis and crowned dens syndrome are rare pathologies also characterized by an acute onset of neck pain, neck stiffness, and fever (7–10). Moreover, inflammatory markers such as serum C-reactive protein (CRP) and erythrocyte sedimentation rate (ESR) are increased in both these diseases, as in meningitis (7–10). Since the symptoms of these two diseases are similar enough to those of meningitis, diagnosis can be difficult if background knowledge of these two diseases is lacking. Here, we report one case each of acute calcific retropharyngeal tendinitis and crowned dens syndrome and discuss the salient points for identifying each disease.

## Case reports

### Case 1

**Figure 1:**
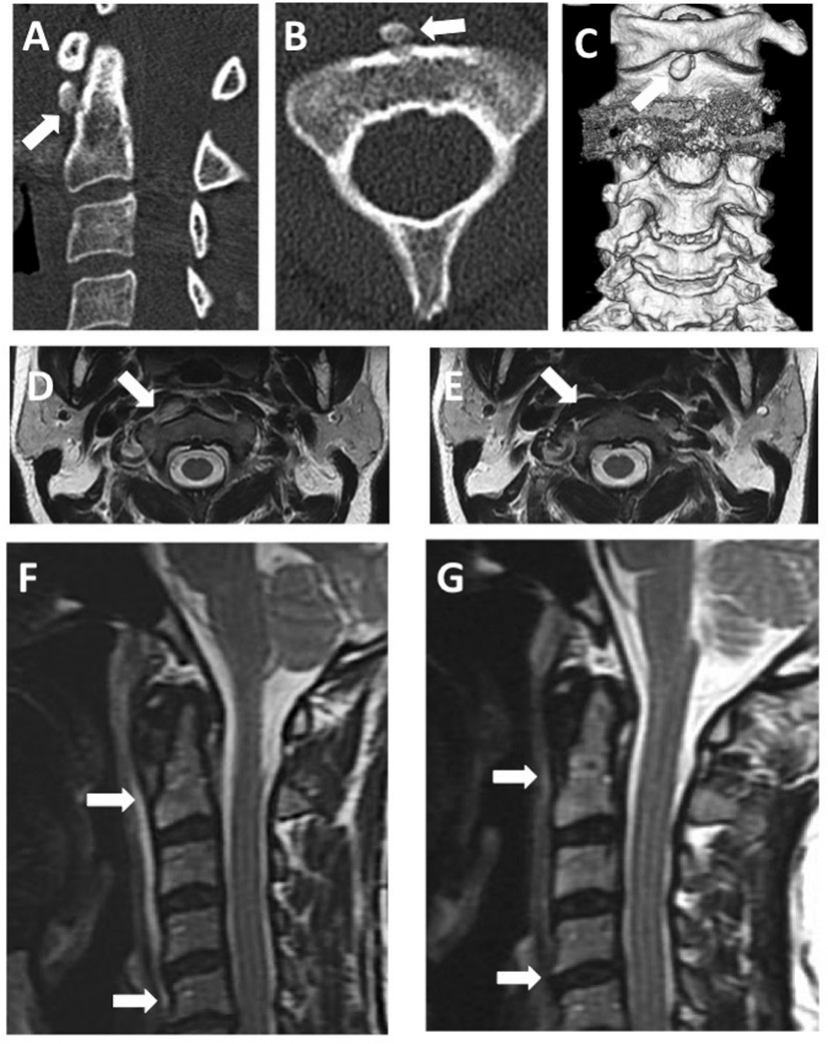
Images of acute calcific retropharyngeal tendinitis. **(A)** Sagittal cervical CT. **(B)** Axial cervical CT. **(C)** Three-dimensional reconstruction of cervical CT images. All cervical CT scans reveal nodular calcification in front of the C1–C2 vertebra (arrow). **(D,F)** T2-weighted cervical MRI before treatment. **(E,G)** T2-weighted cervical MRI after treatment. Before treatment, MRI reveals swelling and prevertebral edema (arrows). After treatment, swelling and edema have disappeared.

A 56-year-old woman presented with a 2-day history of pain in the posterior neck. Neck pain progressively worsened along with limitations to neck movement, and body temperature increased to 38.0°C. Although her body temperature decreased slightly on ice, she visited our hospital's emergency room after neck pain progressed to a headache. Her past medical history was unremarkable, and she had never experienced similar neck pain. On admission, vital signs were blood pressure, 149/79 mmHg; heart rate, 84 beats/min; body temperature, 37.4°C; and respiratory rate, 14 breaths/min. A general physical examination showed no cervical lymphadenopathy. In terms of consciousness, the patient was alert and oriented. A neurological examination showed no abnormalities, with no motor or sensory symptoms or deficits. As for meningeal signs, neck stiffness was present, but both Kernig's and Brudzinski's signs were absent. The patient exhibited odynophagia, tenderness over the posterior neck, and an associated decrease in the range of neck motion in all directions. Laboratory findings were leukocyte count, 9.100/μl (reference: 3.900–9.700/μl); red blood cell count, 4.67 × 10^6^/μl (reference: 3.80–5.04 × 10^6^/μl); platelet count, 205 × 10^3^/μl (reference: 153–346 × 10^3^/μl); total protein, 6.3 g/dl (reference: 6.5–8.5 g/dl); and CRP, 0.68 mg/dl (reference: 0.0–0.29 mg/dl). A lumbar puncture was performed due to severe neck stiffness and headache. Cerebrospinal fluid (CSF) examination showed: cell count, 1 cell/μl (reference: <5 cell/μl); protein, 28 mg/dl (reference: 15–45 mg/dl); and glucose, 68 mg/dl (reference: 50–75 mg/dl). CSF cultures yielded no bacterial growth. Cervical computed tomography (CT) identified a nodular calcification in front of the second cervical vertebra (Figures 1A–C), and cervical magnetic resonance imaging (MRI) showed a hyperintense signal in the right longus colli muscle on T2-weighted imaging (Figures 1D,F).

Acute calcific retropharyngeal tendinitis was diagnosed based on the calcification in front of the second cervical vertebra. The patient could not move her body because of neck pain and was unable to eat meals because of pain in swallowing. The following day, serum CRP levels increased to 8.00 mg/dl, and intravenous acetaminophen was, therefore, started for pain control. On day three, after symptom onset, neck pain decreased slightly, but she still could not move her neck. Odynophagia decreased, and she became able to swallow both drugs and food. On day five, after symptom onset, the pain subsided to the degree that she could move her neck. The pain completely resolved 10 days after its onset, and she was discharged from the hospital. One month after symptom onset, a cervical spine MRI showed no abnormalities in the longus colli muscle (Figures 1E,G). The follow-up after 2 years showed no recurrence of symptoms and no need for further treatment.

### Case 2

**Figure 2:**
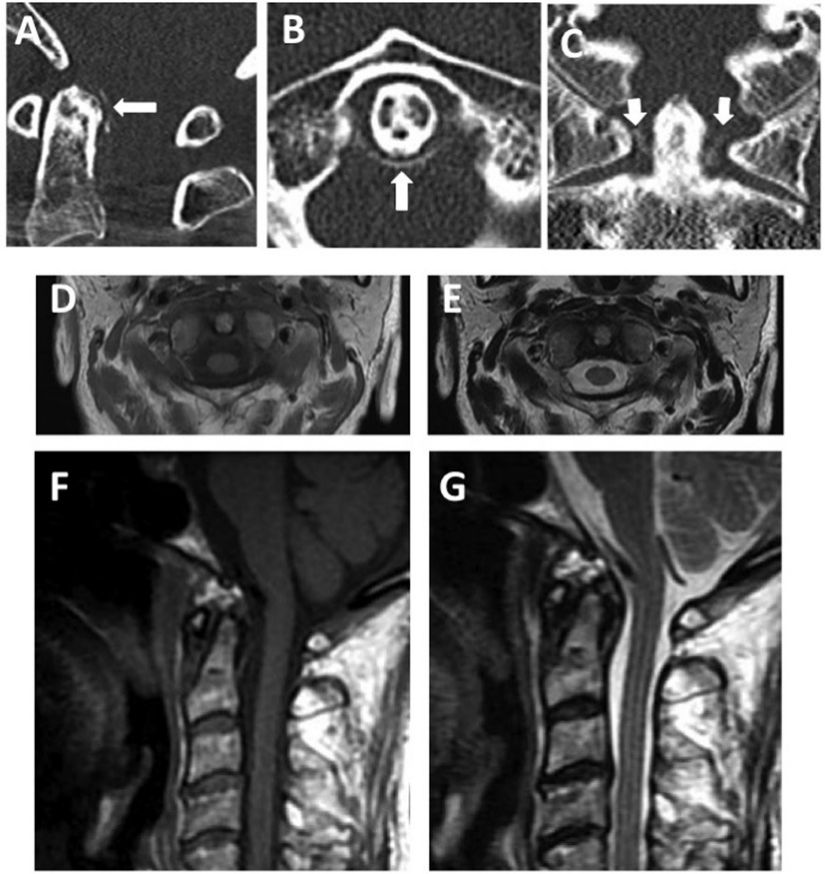
Images of crowned dens syndrome. **(A)** Cervical sagittal CT. **(B)** Cervical axial CT. **(C)** Cervical coronal CT. All cervical CT scans reveal calcified lesions around the dens. **(D–G)** Cervical T1-weighted imaging [**(D)** axial; **(F)** sagittal] and T2-weighted imaging [**(E)** axial; **(G)** sagittal] before treatment. A cervical MRI reveals no swelling or prevertebral edema.

A 75-year-old woman visited our emergency department with neck pain and an occipital headache she had never experienced before. The headache was accompanied by a high fever (up to 38.0°C), so she visited the emergency room of our hospital. Her past medical history was unremarkable. On admission, vital signs were blood pressure, 118/70 mmHg; heart rate, 75 beats/min; body temperature, 37.6°C; and respiratory rate, 18 breaths/min. A general physical examination revealed no abnormalities. In terms of consciousness, she was alert and oriented. A neurological examination showed no abnormalities. She had neck stiffness but negative results for both Kernig's and Brudzinski's signs. Laboratory findings included leukocyte count, 5.800/μl (reference: 3.900–9.700/μl); red blood cell count, 3.28 × 10^6^/μl (reference: 3.80–5.04 × 10^6^/μl); platelet count, 201 × 10^3^/μl (reference: 153–346 × 10^3^/μl); total protein, 6.6 g/dl (reference: 6.5–8.5 g/dl); and CRP, 9.21 mg/dl (reference: 0.0–0.29 mg/dl). We suspected meningitis based on the severe headache, neck stiffness, and increasing CRP. CSF examination showed cell count, 1 cell/μl (reference: <5 cell/μl); protein, 55.8 mg/dl (reference: 15–45 mg/dl); and glucose, 63 mg/dl (reference: 50–75 mg/dl). CSF cultures yielded no bacterial growth. Despite significant neck pain, laboratory findings did not support the presence of meningitis. Cervical CT showed a curvilinear peri-odontoid calcification in the transverse ligament of the atlas on an axial view (Figures 2A–C). Cervical MRI showed normal signal intensity in the longus colli muscle on T2-weighted imaging (Figures 2D–G). Based on these imaging findings, we diagnosed crowned dens syndrome.

On admission, the patient could not move her body because of neck pain. Oral administration of non-steroidal anti-inflammatory drugs (NSAIDs) was started for pain control. The neck pain decreased 3 days after its onset, and she was able to walk unaided. Nine days after symptom onset, neck pain had almost completely resolved, serum levels of CRP had decreased to 1.17 mg/dl, and the patient was discharged from the hospital. Follow-up after 3 years showed no recurrence of symptoms and no further treatment.

## Discussion

Acute calcific retropharyngeal tendinitis is characterized by the sudden onset of acute neck pain, as first reported by Hartly in 1964 (7). This pathology of inflammation in the tendon of the longus colli muscle is triggered by the deposition of calcium hydroxyapatite crystals (11). Although the mechanisms underlying calcification and inflammation remain unclear, ischemic or necrotic changes in the tendons caused by repeated exercise are potential risk factors for calcification (11, 12). This disease is rare, with an estimated incidence of 0.5 cases per 100,000 person-years (13). Acute calcific retropharyngeal tendinitis is most common between the ages of 30 and 60 years, with no obvious sex predominance (8, 14). Previous reports have revealed three major symptoms of neck pain, stiffness, and odynophagia (8, 13–15). Other symptoms that occasionally occur with this disease include shoulder pain, arm pain, back pain, headache, dizziness, nausea, and mild-to-moderate fever (8). In terms of laboratory data, white blood cell counts range from normal to mild leukocytosis, and levels of CRP and ESR are increased in most cases (8, 13–15). As in our case, CSF findings are typically within normal limits (16). Cervical CT is useful for diagnosing acute calcific retropharyngeal tendinitis, showing increased soft-tissue shadows ventral to the cervical spine and calcification anterior to the C1/2 vertebra. Cervical MRI shows diffuse swelling of the longus colli muscle and signals hyperintensity on T2-weighted imaging. Retropharyngeal calcific tendinitis is a self-limiting condition, with symptoms usually resolving spontaneously within 1–2 weeks. Acute calcific retropharyngeal tendinitis responds well to NSAIDs, with or without corticosteroids, and neck pain usually decreases within a few days after starting such treatment (8, 13–15). Immobilization with a soft cervical collar is another useful method to avoid the aggravation of symptoms (11).

Crowned dens syndrome is caused by pseudogout of the atlantoaxial junction due to the deposition of calcium pyrophosphate dehydrate or calcium hydroxyapatite around the dens. This pathology was first described in 1985 by Bouvet et al. (9) and Godfrin-Valnet et al. (17). The incidence of crowned dens syndrome is unclear, although the condition reportedly accounts for 1.9% of outpatients complaining of neck pain (10). Crowned dens syndrome tends to occur in older women, particularly those over 60 (18). Previous reports have revealed three common symptoms: neck pain, neck stiffness, and fever (10). Shoulder pain, occipital pain, pharyngalgia, myelopathy, vomiting, and jaw claudication are occasionally seen in this disease. In terms of laboratory data, white blood cell counts range from normal to mild leukocytosis, while levels of CRP and ESR are increased in most cases (10, 18). Findings from CSF are within normal limits (19–21). Cervical CT is useful for diagnosing, showing linear calcification around the dens. These findings represent the “gold standard” for diagnosis, with a diagnosis rate of 97.1% (10). The treatment of crowned dens syndrome involves the administration of NSAIDs, usually leading to symptom resolution within a few days to weeks. Steroids are also effective in severe or recurrent cases (10).

Both acute calcific retropharyngeal tendinitis and crowned dens syndrome commonly present with severe neck pain and fever. The common presence of neck stiffness, fever, and headache in some patients can lead clinicians to misdiagnose meningitis. A few key points distinguish acute calcific retropharyngeal tendinitis and crowned dens syndrome from meningitis. First, according to one report, a limitation of neck rotation to < 45° suggests the involvement of the C1/2 joint (21). Crowned dens syndrome is caused by pseudogout of the atlantoaxial junction. On the other hand, the upper part of the longus colli muscle arises from C3–C5 to the atlas and is associated with neck flexion and contralateral rotation. Therefore, both crowned dens syndrome and acute calcific retropharyngeal tendinitis involve the C1/2 joint. When encountering a patient with neck pain and severe limitation of neck movement, clinicians should consider acute calcific retropharyngeal tendinitis and crowned dens syndrome as possibilities and perform cervical CT. Second, altered consciousness is common in cases of meningitis (6) but not in cases of acute calcific retropharyngeal tendinitis or crowned dens syndrome (7–20). When encountering a patient with neck pain and altered consciousness, clinicians should consider the possibility of meningitis and perform a CSF examination. In patients for whom diagnosis proves difficult, CSF examination and cervical CT are useful for distinguishing meningitis from acute calcific retropharyngeal tendinitis or crowned dens syndrome (Table 1).

**Table 1:** Clinical features of each disease.

	**Meningitis**	**Acute calcific retropharyngeal tendinitis**	**Crowned dens syndrome**
Age/sex	All ages	Middle-age	High age (>60 years old)
	Difference by causes	Men ≒ women	Men < women
Examination	White blood cells ↑	White blood cells ↑	White blood cells ↑
	CRP ↑	ESR, CRP ↑	ESR, CRP↑
	Cerebrospinal fluid cells ↑	Cerebrospinal fluid cells normal	Cerebrospinal fluid cells normal
Symptoms	Headache	Neck pain	Neck pain
	Fever (> 38°C)	Fever	Fever
	Photophobia	Neck stiffness	Neck stiffness
	Vomiting	Odynophagia	Kernig's sign (−)
	Neck stiffness	Kernig's sign (−)	Brudzinski's sign (−)
	Altered consciousness	Brudzinski's sign (−)	
Cervical CT Findings	Normal	Increased soft tissue shadow ventral to cervical spine and calcifications anterior to C1/2 vertebra	Linear calcifications around the dens
Treatment	Acyclovir	NSAIDs	NSAIDs
	Antibiotics	Steroid	Steroid
Prognosis	Varied	Recovery within 1–2 weeks	Recovery within 1–2 weeks

CRP, C-reactive protein; CT, computed tomography; ESR, erythrocyte sedimentation rate; NSAIDs, non-steroidal anti-inflammatory drugs; ↑, increase; (−), absent.

Differentiating acute calcific retropharyngeal tendinitis from crowned dens syndrome is also important. Crowned dens syndrome tends to be more common among older women, whereas acute calcific retropharyngeal tendinitis most often appears during middle age (Table 1). Odynophagia appears in more than 80% of patients with acute calcific retropharyngeal tendinitis (8), while pharyngalgia appears in only 8.3% of patients with crowned dens syndrome (10). In fact, odynophagia might be relatively specific to acute calcific retropharyngeal tendinitis. As in acute calcific retropharyngeal tendinitis, neck CT is the best tool for diagnosing crowned dens syndrome. Our experience suggests that when examining patients with neck pain and fever, the possibilities of acute calcific retropharyngeal tendinitis and crowned dens syndrome should always be considered, using cervical CT to make a definitive diagnosis. An appropriate diagnosis is needed to avoid unnecessary invasive treatments or inappropriate administration of antibiotics.

## Data availability statement

The original contributions presented in the study are included in the article/supplementary material, further inquiries can be directed to the corresponding author.

## Ethics statement

Written informed consent was obtained from the individual(s) for the publication of any potentially identifiable images or data included in this article.

## Author contributions

EI performed the data research and wrote the manuscript. EI, JF, and KK treated the patients. AF, AN, and HE supported the clinical interpretation. NH and YS were critically involved in the theoretical discussion and composition of the manuscript. All authors read and approved the final version of the manuscript.

## Funding

Author AN was funded by grants from the Japan Society for the Promotion of Science and a Grant-in-Aid for Scientific Research (21K15751). Author NH was funded by grants from the Japan Society for the Promotion of Science (JSPS), Japan Agency for Medical Research and Development (AMED), and Ministry of Education Culture, Sports, Science and Technology Japan; Grant-in-Aid for Scientific Research (21H04820). Author YS was funded by grants from the Japan Society for the Promotion of Science and a Grant-in-Aid for Scientific Research (21K07282).

## Conflict of interest

Author NH received speaker or advisory board honoraria from Kyowa Kirin, Takeda Pharma, AbbVie GK, Sumitomo Dainippon Pharma, Eisai, Mochida Pharma, Kissei Pharma, Ono Pharma, Teijin Pharma, Senju Pharma, EA Pharma, and Novartis Pharma K.K. He also received consulting honoraria from Mitsubishi Tanabe Pharma, Hisamitsu Pharma, and Chugai Pharma. Author YS received speaker honoraria from Medtronic, Boston Scientific, Otsuka Pharmaceutical, Takeda Pharmaceutical Co., Sumitomo Dainippon Pharma, Novartis Pharma, MSD, FP Pharmaceutical Corporation, Kyowa Hakko Kirin, and AbbVie, Inc. The remaining authors declare that the research was conducted in the absence of any commercial or financial relationships that could be construed as a potential conflict of interest.

## Publisher's note

All claims expressed in this article are solely those of the authors and do not necessarily represent those of their affiliated organizations, or those of the publisher, the editors and the reviewers. Any product that may be evaluated in this article, or claim that may be made by its manufacturer, is not guaranteed or endorsed by the publisher.
